# Parental Warmth and Hostility and Child Executive Function Problems: A Longitudinal Study of Chinese Families

**DOI:** 10.3389/fpsyg.2018.01063

**Published:** 2018-07-04

**Authors:** Chun Bun Lam, Kevin Kien Hoa Chung, Xiaomin Li

**Affiliations:** Department of Early Childhood Education, The Education University of Hong Kong, Tai Po, Hong Kong

**Keywords:** Chinese families, early childhood, executive functions, longitudinal, parenting

## Abstract

This study examined the longitudinal associations of maternal and paternal warmth and hostility with child executive function problems. Data were collected for two consecutive years from 333 kindergarten children who resided in Hong Kong, China, as well as their mothers, fathers, and class teachers. At Time 1, the average age of children was 57.73 months, and 56% of them were girls. At Time 1, mothers and fathers rated their own parenting practices with their children. At Times 1 and 2, class teachers rated children’s problems in three aspects of executive functions, including updating/working memory, inhibition, and shifting/cognitive flexibility. As control variables, at Time 1, parents provided information on child and family demographic factors, and children completed verbal ability tasks. Multilevel modeling revealed that controlling for child and family demographic factors, child verbal abilities, and paternal parenting practices, maternal hostility, but not maternal warmth, was linked to increases in child inhibition and shifting/cognitive flexibility problems. Moreover, paternal hostility, but not paternal warmth, was linked to increases in updating/working memory problems. Theoretically, this study highlighted the importance of considering the contributions of both mothers and fathers, and differentiating between positive and negative aspects of parenting, when examining the development of child executive functions. Practically, this study pointed to the utility of targeting maternal and paternal hostility in family intervention and community education in order to reduce child executive function problems.

## Introduction

Emerging research indicates that children’s executive functions, or abilities to regulate thoughts and behaviors to achieve desirable goals (Garon et al., 2008; Best and Miller, 2010; Miyake and Friedman, 2012; Diamond, 2013), have important implications for their adjustment. Children with better executive functions, for example, are more popular among peers, exhibit fewer conduct problems, and perform better in school exams and standardized tests (Ellis et al., 2009; Chung and McBride-Chang, 2011; Jacobson et al., 2011; Chung et al., 2018). Given the importance of executive functions, researchers have been examining how such outcomes are affected by different personal (e.g., brain development and physical activity) and environmental (e.g., family and school experiences) factors (Hughes, 2011; Diamond, 2014). An emerging line of work focuses specifically on parental influences (Fay-Stammbach et al., 2014). Most of this work, however, has relied on cross-sectional data, focused on the roles of mothers, and measured either positive or negative aspects of parenting. Moreover, nearly all studies on parenting and child executive functions are based on European, Canadian, or European American samples. Although about one-fifth of the world population lives in China (United Nations, 2017), we know next to nothing about how the family may affect child executive functions in Chinese families. Grounded in the theories of attachment (Shaver and Mikulincer, 2010; Bernier et al., 2012) and social learning (Hughes and Ensor, 2009; Moriguchi, 2012, 2014), the present study examined the longitudinal associations of maternal and paternal warmth and hostility with child executive function problems in a sample of kindergarten children from Hong Kong, China. We focused on early childhood, as younger children are more dependent on their caretakers for protection, nurturance, and simulation, and thus may be more susceptible to parental influences (Halgunseth, 2009).

### Theoretical Perspectives on Parenting and Child Executive Functions

Executive functions refer to individuals’ abilities to override more automatic thoughts and responses, and behave in planned, goal-directed manners, especially in novel or ambiguous situations (Diamond, 2013). Executive functions involve three subcomponents (Best and Miller, 2010; Miyake and Friedman, 2012), namely *updating/working memory*, or the ability to hold and manipulate information in working memory to direct behaviors toward future goals, *inhibition*, or the ability to suppress unthinking or inappropriate thoughts and responses, and *shifting/cognitive flexibility*, or the ability to shift focus to the mental framework most relevant to the task at hand and choose from potentially conflicting behavioral alternatives. Although distinguishable, the three subcomponents of executive functions often operate in integrated ways to support higher order processing. According to an integrative model of executive functions (Garon et al., 2008), for example, updating/working memory, which directs attention to relevant information, inhibition, which directs attention away from irrelevant information, and shifting/cognitive flexibility, which shifts attention depending on the demand of the situation, are all central to the management of oneself, completion of tasks, and adjustment to challenges. Not surprisingly, children with better executive functions score higher on a wide range of adjustment indices, including peer competence, behavioral conduct, and academic achievement (Ellis et al., 2009; Chung and McBride-Chang, 2011; Jacobson et al., 2011; Chung et al., 2018).

Multiple theories have been used to explain how parenting may affect child executive functions. The attachment theory, for example, posits that secure relationships with caretakers may “free up” cognitive resources for self-directed exploration of the environment, which involves playing with physical objects and interacting with nonfamilial members (Shaver and Mikulincer, 2010; Bernier et al., 2012). Specifically, securely attached children, who perceive their caretakers as reliable sources of protection and support, spend fewer cognitive resources monitoring their caretakers’ availabilities and worrying about being abandoned. The cognitive resources freed up can be instead invested in self-directed exploration of the environment, resulting in additional opportunities for these children to practice their executive functions. Therefore, parenting behaviors that facilitate and hinder the formation of secure attachment may facilitate and hinder the development of child executive functions, respectively.

On the other hand, the social learning theory posits that children tend to imitate others’ behaviors, especially the behaviors of their parents, whom they are close to and dependent on (Hughes and Ensor, 2009; Moriguchi, 2012, 2014). In fact, children’s tendency to imitate their parents is so strong that children may remember and reproduce their parents’ behaviors, even when these behaviors are not linked to any rewards in the first place. Once learnt, these behaviors may also be highly resistant to changes, even when met with punishment. Therefore, parenting behaviors that model good and poor self-regulation may have positive and negative impacts on the development of child executive functions, respectively.

Echoing the theories of attachment and social learning, Blair et al. (2011) emphasized the importance of considering both positive and negative aspects of parenting when studying it as a potential correlate of child executive functions. Parental warmth, which involves parental support, praises, and displays of affection and tranquility, may model good self-regulation, lead to more positive parent–child exchanges, and promote children’s executive function skills. Meanwhile, parental hostility, which involves parental rejection, reprimands, and loss of temper and control, may model poor self-regulation, lead to more negative parent–child interactions, and create children’s executive function problems. A comprehensive review on family influences on the development of child executive functions can be found in Fay-Stammbach et al. (2014).

### Empirical Findings on Parenting and Child Executive Functions

Empirical findings on parenting and child executive functions are generally consistent with predictions of the theories of attachment and social learning. However, as we elaborate below, existing research has been mostly based on cross-sectional data, focused on either positive or negative aspects of parenting, and highlighted the contributions of mothers. What is lacking in the literature is longitudinal research that controls for prior levels of child executive functions, includes both parental warmth and hostility in the same analytic model, and is based on data from both mothers and fathers.

Numerous cross-sectional studies have showed that parental warmth and hostility are associated positively and negatively with child executive function skills, respectively (Hughes and Ensor, 2009; Schroeder and Kelley, 2010; Hopkins et al., 2013; Linebarger et al., 2014; Sosic-Vasic et al., 2017). Longitudinal work is rarer, but several studies showed that maternal warmth, measured in infancy and toddlerhood, was positively linked to child executive function skills (Matte-Gagné et al., 2015; Meuwissen and Englund, 2016) and negatively linked to child executive function problems, measured in early childhood (Kraybill and Bell, 2013). One study further showed that both positive and negative aspects of maternal parenting, measured in infancy and toddlerhood and included in the same analytic model, were uniquely predictive of children’s overall skills of updating/working memory, inhibition, and shifting/cognitive flexibility, measured in early childhood (Blair et al., 2011).

Longitudinal findings controlling for prior levels of child executive functions are more mixed. For example, in Cuevas et al.’s (2014) study, maternal hostility measured when the child was 10-, 24-, and 36-month old was negatively linked to child executive functions measured when the child was 48-month old, even after controlling for child executive functions measured when the child was 36-month old. Moreover, in Devine et al.’s (2016) study, maternal warmth and hostility, measured when the child was 48-month old and included in the same analytic model, were uniquely predictive of child executive function measured when the child was 60-month old, even after controlling for child executive functions measured when the child was 48-month old. However, in Bernier et al.’s (2010) study, the association between maternal warmth (measured when the child was 18-month old) and child executive functions (measured when the child was 26-month old) became nonsignificant, after controlling for child executive functions measured when the child was 18-month old. Taken together, these findings – all of which were based on children’s overall skills of updating/working memory, inhibition, and shifting/cognitive flexibility – highlighted the need of additional research that examines the unique roles of parental warmth and hostility in understanding changes in child executive functions over time.

Another need of additional research concerns the roles of fathers. Despite increasing evidence indicating that paternal parenting practices are uniquely linked to child adjustment (Lam and McHale, 2012; Lam et al., 2012; Cabrera et al., 2014), only a handful of studies have examined the potential contributions of fathers to their children’s development in executive functions. For example, in one cross-sectional study (Meuwissen and Carlson, 2015), paternal warmth and hostility were linked positively and negatively to children’s overall skills of updating/working memory, inhibition, and shifting/cognitive flexibility, respectively. In another cross-sectional study (Lucassen et al., 2015), with both positive and negative aspects of maternal and paternal parenting being included in the same analytic model, paternal hostility (but not paternal warmth) was linked to child problems in updating/working memory and inhibition (but not shifting/cognitive flexibility). Finally, in a longitudinal study (Towe-Goodman et al., 2014), maternal and paternal warmth, measured when the child was 24-month old and included in the same analytic model, were uniquely linked to child overall skills of updating/working memory, inhibition, and shifting/cognitive flexibility, measured when the child was 36-month old. To our best knowledge, no studies have tested whether the warmth and hostility of both mothers and fathers, *all included in the same analytic model*, are uniquely linked to *changes* in children’s executive functions over time.

It is worth mentioning that executive functions, especially in the form of inhibition, are highly emphasized in Hong Kong (Kwong et al., 2018), as well as other Chinese communities (Chen et al., 2012). For example, Chinese parents expect their children to master inhibition as early as toddlerhood, but US parents do not expect their children to do so until early childhood (Stevenson et al., 1990; Chen et al., 1998). Also, in kindergartens, Chinese children are expected to stay more focused on tasks, comply with more instructions, and sit still for longer periods of time compared to US children (Tobin et al., 2009). Probably because of these culturally unique expectations, Chinese children mature more quickly in executive functions than do their Western counterparts (Sabbagh et al., 2006; Ellefson et al., 2017). Despite such a strong emphasis on self-regulation early on in Chinese communities, existing research on child executive functions was largely based on European, Canadian, or European American families. We know next to nothing about whether Chinese mothers’ and fathers’ warmth and hostility may affect their children’s executive functions. By examining such research questions in a sample of Chinese families, we sought to move beyond the typical focus on Western families to shed light on the role of parenting in the development of children’s executive functions in the Eastern part of the world.

### The Present Study

To recap, existing research on parenting and child executive functions tends to rely on cross-sectional data, measure either positive or negative aspect of parenting, and focus on the roles of mothers. Guided by the theories of attachment (Shaver and Mikulincer, 2010; Bernier et al., 2012) and social learning (Hughes and Ensor, 2009; Moriguchi, 2012, 2014), the present study addressed these gaps in the literature by linking maternal and paternal warmth and hostility (separately reported by the mother and the father when the child was 58-month old) to changes in child executive function problems over time (independently reported by the kindergarten class teacher when the child was 58- and 70-month old). To model longitudinal changes, we used child executive function problems at Time 2 as the dependent variables and included child executive function problems at Time 1 as controls (Rovine and Liu, 2012). We further controlled for child gender and age and maternal and paternal education levels, as well as child verbal abilities, as an indicator of child verbal or crystalized intelligence (Norton and Wolf, 2012; Schipolowski et al., 2014), in order to isolate the impact of parenting on child executive functions from those of child and family demographic factors (Diamond, 2013; Fay-Stammbach et al., 2014) and child general cognitive functioning (Bernier et al., 2010; Cuevas et al., 2014; Towe-Goodman et al., 2014).

## Materials and Methods

### Participants and Procedures

Participants were 333 children, as well as their mothers, fathers, and class teachers, from 10 kindergartens in Hong Kong. Kindergartens in Hong Kong varied highly in size, with the number of students in each kindergarten ranging from 100 to 800. To ensure that families from diverse socioeconomic backgrounds were recruited, we used a stratified sampling approach: Based on their median monthly household incomes (Census and Statistics Department, 2015), we first stratified the 18 geographic districts of Hong Kong into high, middle, and low socioeconomic strata. We then randomly called kindergartens (using publicly available contact information) until three kindergartens in each stratum agreed to recruit families for the study. Two kindergartens recruited from the high socioeconomic stratum turned out to be small in size. Therefore, we recruited one more kindergarten from that stratum to strive for a more balanced distribution of families from different socioeconomic backgrounds. We sent invitation letters and consent forms to all second-year students in the 10 kindergartens. Three hundred and thirty three families provided informed and written consent for us to collect data from the children, as well as the mothers, fathers, and class teachers. The class teachers also provided informed and written consent to participate in the study.

Data collection took place in the second semesters of the academic years of 2014–2015 (Time 1) and 2015–2016 (Time 2), which were separated by about 12 months. At Time 1, mothers and fathers separately rated their own parenting practices using self-administered questionnaires, and provided child and family demographic information. Children also completed tasks on verbal abilities with trained administrators from our research team. At Times 1 and 2, class teachers rated children’s executive function problems using self-administered questionnaires. The retention rate across Times 1 and 2 was 89%.

Maternal and paternal education levels averaged 3.12 (*SD* = 1.15) and 3.14 (*SD* = 1.21), respectively, on a 5-point scale ranging from 1 (*elementary school education*) to 5 (*postgraduate education*). Fifty-six percent of children were girls (*n* = 185). The mean ages of children were 57.73 (*SD* = 4.53) and 70.07 months (*SD* = 4.55) at Times 1 and 2, respectively. At each time point, each parent received a supermarket coupon of HK$50 (or about US$6), and each teacher received a supermarket coupon of HK$100 (or about US$12), after completing the questionnaire. Each child received a gift of HK$5 (or about US$1) after completing the tasks. The present study was approved by the Human Research Ethics Committee of the Education University of Hong Kong.

### Measures

Following Foster and Martinez’s (1995) recommendations, all English measures were forward and backward translated to Chinese by two independent translators, before two local family researchers resolved the discrepancies and finalized the items. The finalized items were further reviewed, discussed, and fine-tuned in a pilot study with 20 parents and 10 teachers of kindergarten children, to ensure the clarity of the questions and instructions.

*Parental warmth* and *hostility* were measured using the 7-item warmth/acceptance and the 3-item verbal hostility subscales from the Parenting Styles and Dimensions Questionnaire (Robinson et al., 2001). At Time 1, on a 5-point scale ranging from 1 (*never*) to 5 (*always*), mothers and fathers rated how often they praised and were affectionate with their children (e.g., “I give praises when this child is good,” “I express affection by hugging, kissing, and holding this child”), and how often they lost temper at and had conflict with their children (e.g., “I explode in anger towards this child,” “I yell or shout when this child misbehaves”). Item ratings were averaged, with higher scores indicating higher levels of warmth and verbal hostility. The reliability and validity of the Parenting Styles and Dimensions Questionnaire had been evidenced in samples of Chinese parents (Wu et al., 2002; Lee E.H. et al., 2013). In the present study, the Cronbach’s alphas of maternal and paternal warmth were 0.85 and 0.84, and those of maternal and paternal verbal hostility were 0.67 and 0.68, respectively.

*Child executive function problems* were measured using the Behavior Rating Inventory of Executive Functions (BRIEF; Gioia et al., 2003; Sherman and Brooks, 2010). We opted to use the BRIEF, as it had been the most commonly used rating scale of executive functions in the literature (Toplak et al., 2012). It included five subscales, including the 10-item emotion regulation, 16-item inhibitory control, 10-item shifting, 17-item working memory, and 10-item planning and organizing subscales. At Times 1 and 2, on a 3-point scale ranging from 1 (*never*) to 3 (*often*), class teachers rated problems that children had in modulating their emotional responses, resisting impulses, and stopping their behaviors at the appropriate time, moving freely from one situation or activity to another, holding information in mind, and managing current and future-oriented task demands. Item ratings were averaged, with higher scores indicating higher levels of executive function problems.

The five subscales of the BRIEF could be summarized as three composite scores, namely emergent metacognition (composed of the working memory and planning and organizing subscales, indicating the ability to initiate, decide, plan, and implement future-oriented problem solving), inhibitory self-control (composed of the emotional control and inhibition subscales, indicating the ability to modulate emotions and responses through inhibition), and flexibility (composed of the emotional control and shifting subscales, indicating the ability to move flexibly among behaviors), which roughly captured the three aforementioned subcomponents of executive functions (i.e., *updating*/*working memory, inhibition*, and *shifting*/*cognitive flexibility*; Lucassen et al., 2015; Garon et al., 2016; Skogan et al., 2016). The reliability and validity of the BRIEF had been evidenced in samples of Chinese children (Chan et al., 2009; Qian et al., 2010). In the present study, at Time 1, the Cronbach’s alphas of emotion regulation, inhibitory control, shifting, working memory, and planning and organizing were 0.89, 0.92, 0.90, 0.95, and 0.90, respectively. At Time 2, the Cronbach’s alphas of emotion regulation, inhibitory control, shifting, working memory, and planning and organizing were 0.92, 0.95, 0.93, 0.96, and 0.94, respectively.

*Child verbal abilities* were measured using a task on rapid automatized naming (Shu et al., 2006). Rapid automatized naming, or the ability to name as fast as possible highly familiar visual stimuli, such as digits, letters, colors, and objects, has been consistently linked to verbal abilities in different cultures (Norton and Wolf, 2012). At Time 1, children were presented with five rows of five digits printed in random order on a piece of paper (e.g., 2, 4, 6, 7, and 9). Children were then asked to read the digits aloud as fast as possible. Each child did the task twice, and the mean time (in seconds) to complete the two trials was computed. Lower scores indicated higher levels of verbal ability. The reliability and validity of the task had been evidenced in samples of Chinese children (Chan et al., 2006; Yeung et al., 2013). In the present study, the test–retest reliability was 0.82.

Other control variables, including child gender and age and maternal and paternal education levels were provided by mothers and fathers at Time 1.

## Results

### Preliminary Analyses

**Table 1** presents the means and standard deviations of all major variables. **Table 2** presents the correlations among them. Focusing on the longitudinal associations between parenting and child executive functions, maternal warmth at Time 1 was negatively correlated with child updating/working memory problems at Time 2. Maternal hostility at Time 1 was positively correlated with child updating/working memory, inhibition, and shifting/cognitive flexibility problems at Time 2. Paternal warmth at Time 1 was negatively correlated with child updating/working memory, inhibition, and shifting/cognitive flexibility problems at Time 2. Paternal hostility at Time 1 was positively correlated with child updating/working memory problems at Time 2. It is worth noting that child updating/working memory, inhibition, and shifting/cognitive flexibility problems were strongly and positively correlated with one another at both time points. They also showed moderate stability across Times 1 and 2.

**Table 1:** Descriptive statistics of all key variables.

	*M*	*SD*	Range
Maternal warmth (T1)	4.19	0.49	2.54–5.00
Maternal hostility (T1)	2.41	0.59	1.00–5.00
Paternal warmth (T1)	3.91	0.54	2.00–5.00
Paternal hostility (T1)	2.34	0.60	1.00–4.00
Child U/WMP (T1)	1.46	0.41	1.00–2.76
Child IP (T1)	1.37	0.34	1.00–2.63
Child S/CFP (T1)	1.33	0.34	1.00–2.40
Child U/WMP (T2)	1.32	0.42	1.00–2.94
Child IP (T2)	1.26	0.35	1.00–2.86
Child S/CFP (T2)	1.20	0.32	1.00–2.95
Child gender^a^	0.56	0.50	0.00–1.00
Child age (T1)	57.73	4.53	50.00–83.00
Child verbal abilities (T1)	24.14	8.54	10.40–75.59
Maternal education (T1)	3.12	1.15	1.00–5.00
Paternal education (T1)	3.14	1.20	1.00–5.00

*U/WMP, updating/working memory problems; IP, inhibition problems; S/CFP, shifting/cognitive flexibility problems; VA, verbal abilities; EDU, education; T1, Time 1; T2, Time 2. ^*a*^Coded as 0 = boy and 1 = girls; 56% of children were girls.*

**Table 2:** Pearson correlations among all key variables.

Variables	1	2	3	4	5	6	7	8	9	10	11	12	13	14	15
1. M warmth (T1)	–														
2. M hostility (T1)	–0.10	–													
3. P warmth (T1)	0.40**	–0.07	–												
4. P hostility (T1)	–0.16**	0.37**	–0.16**	–											
5. C U/WMP (T1)	–0.16**	0.07	0.04	0.09	–										
6. C IP (T1)	–0.13**	0.16**	–0.05	0.12*	0.74**	–									
7. C S/CFP (T1)	–0.09	0.04	–0.00	0.05	0.64**	0.87**	–								
8. C U/WMP (T2)	–0.19**	0.15**	–0.16**	0.12*	0.43**	0.43**	0.30**	–							
9. C IP (T2)	–0.09	0.20**	–0.16**	0.10	0.27**	0.46**	0.30**	0.81**	–						
10. C S/CFP (T2)	–0.04	0.14*	–0.13*	0.05	0.22**	0.39**	0.32**	0.72**	0.90**	–					
11. C gender^a^	0.14*	–0.11*	0.21**	–0.08	–0.19**	–0.16**	–0.12*	–0.27**	–0.21**	–0.15*	–				
12. C age (T1)	0.00	0.06	0.15**	–0.06	–0.15**	–0.08	–0.08	–0.10	–0.06	–0.04	0.11	–			
13. C VA (T1)	–0.15**	0.09	–0.09	0.04	0.31**	0.20**	0.16**	0.18**	0.05	0.05	–0.14*	–0.13*	–		
14. M EDU (T1)	0.32**	–0.09	0.19**	–0.05	–0.26**	–0.18**	–0.16**	–0.24**	–0.12*	–0.05	0.19**	0.05	–0.27**	–	
15. P EDU (T1)	0.25**	–0.06	0.19**	–0.08	–0.15**	–0.15**	–0.10	–0.12*	–0.03	0.01	0.04	–0.02	–0.23**	0.61**	–

*M, maternal; P, paternal; C, child; U/WMP, updating/working memory problems; IP, inhibition problems; S/CFP, shifting/cognitive flexibility problems; VA, verbal abilities; EDU, education; T1, Time 1; T2, Time 2. ^*a*^Coded as 0 = boy and 1 = girl; indicating point-biserial correlations. ^∗^*p* < 0.05. ^∗∗^*p* < 0.01.*

Before conducting the main analyses, we examined potential attrition bias by comparing families that provided data at both time points versus families that dropped out after Time 1 (Miller and Wright, 1995). Independent sample *t*-tests indicated that, at Time 1, the two groups did not differ in maternal or paternal warmth or hostility, or child updating/working memory, inhibition, or shifting/cognitive flexibility problems (*t*s = -0.15–1.07; *n.s.*). With respect to the control variables, independent sample *t*- and χ^2^ tests indicated that, at Time 1, the two groups did not differ in child age or verbal abilities (*t*s = 0.32 and 0.98, respectively; *n.s.*), or child gender composition (χ^2^ = 1.00; *n.s.*). The two groups did differ in maternal (*t* = -2.19; *p* < 0.05) and paternal (*t* = -2.42; *p* < 0.05) education levels, suggesting that families that dropped out after Time 1 had more educated parents. Therefore, as we detail below, full information maximum likelihood (FIML) was used to correct for potential biases in parameter and standard error estimation in our analyses (Schafer, 1997).

### Main Analyses

Using SAS 9.3, we ran separate multilevel models for child updating/working memory, inhibition, and shifting/cognitive flexibility problems at Time 2. In many ways, multilevel models are similar to the more commonly used, least-square multiple regression models (Raudenbush and Bryk, 2002; Bickel, 2007). But, one major difference lies in their assumptions about the dependence of the individual cases being analyzed: Multiple regression models assume the cases to be unrelated to one another in terms of the dependent variables, whereas multilevel models do not. Given that the same teacher provided ratings on the dependent variables for multiple children in our sample, the case independence assumption of multiple regression models was unlikely to hold. Therefore, we analyzed our data using multilevel models, which allowed the cases to be related to one another by specifying a correlation matrix among the error residuals. As noted, FIML, which treats the mean and variance of the observed cases as parameters, and estimates particular parametric values that would make the overall results the most probable (Schafer, 1997), was used to accommodate our missing data. FIML, along with multiple imputations, is often recommended as the best ways of dealing with nonrandom missing data in multiple regression models (Newman, 2003; Schlomer et al., 2010). However, recent simulation studies have indicated that, in multilevel models with nonrandom missing data, FIML is more effective than multiple imputations in correctly estimating standard errors (Larsen, 2011; Shin et al., 2017).

**FIGURE 1:**
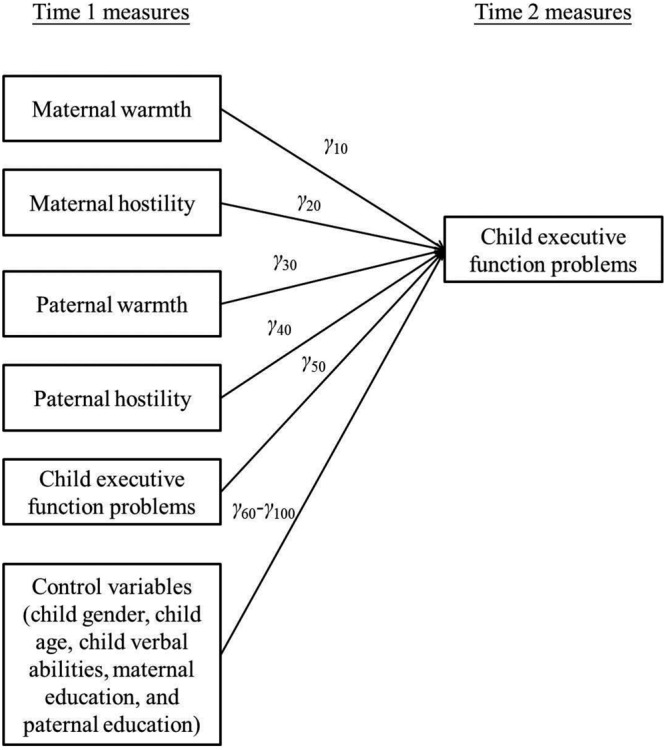
Path diagram representing the analytic model of child executive function problems.

To examine the unique impact of mothers and fathers, we included maternal and paternal warmth and hostility at Time 1 in the same analytic models. Moreover, to model the changes in child executive functions over time, we controlled for corresponding child executive function problems at Time 1 (Rovine and Liu, 2012). Finally, to rule out other child and family factors as alternative explanations, we controlled for child gender, age, and verbal abilities, and maternal and paternal education levels. **Appendix** presents the equations of our multilevel models. **Figure 1** shows the path diagram of the analytic models.

**Table 3** presents the parameter coefficients of our multilevel models. Like the unstandardized coefficients in multiple regression models (*B*), the gamma coefficients in multilevel models (γ) indicate how many units of the dependent variable will change per one unit increase in the predictor variables. Our results indicated that maternal hostility was uniquely linked to *eases* in child inhibition problems and *increases* in child shifting/cognitive flexibility problems. In other words, children exposed to more hostility from their mothers showed more inhibition and child shifting/cognitive flexibility problems in the following year. Moreover, paternal hostility was uniquely linked to *increases* in child working memory problems, meaning that children exposed to more hostility from their fathers showed more updating/working memory problems in the following year. Worth mentioning is that prior levels of executive function problems were significant and positive predictors of all three outcome measures. Overall, the models explained 18, 29, and 10% variance in updating/working memory, inhibition, and shifting/cognitive flexibility problems, respectively, representing moderate to large effect sizes (Cohen, 1988).

**Table 3:** Gamma coefficients (γ) and standard errors (SE) of multilevel models of child executive function problems.

	U/WM problems		I problems		S/CF problems	
Variables	γ	SE	γ	SE	γ	SE
Maternal warmth (γ_10_)	0.02	0.04	0.05	0.04	0.06	0.04
Maternal hostility (γ_20_)	0.04	0.04	0.07*	0.03	0.07*	0.03
Paternal warmth (γ_30_)	–0.04	0.04	–0.05	0.03	–0.05	0.03
Paternal hostility (γ_40_)	0.10**	0.04	0.04	0.03	0.04	0.03
Prior corresponding problems (γ_50_)	0.44**	0.06	0.54**	0.06	0.33**	0.05
Child gender (γ_60_)	–0.09*	0.04	–0.05	0.04	–0.04	0.04
Child age (γ_70_)	0.01	0.00	0.00	0.00	0.00	0.00
Child verbal abilities (γ_80_)	0.00	0.00	–0.00	0.00	0.00	0.00
Maternal education (γ_90_)	0.00	0.02	0.01	0.02	0.02	0.02
Paternal education (γ_100_)	–0.00	0.02	0.01	0.02	0.02	0.02
**Variance components**		
Residual (*r_ij_*)	0.10**	0.01	0.07**	0.01	0.07**	0.01
Intercept (*u*_0_*_j_*)	0.04**	0.01	0.02**	0.01	0.02**	0.01

*U/WM, updating/working memory; I, inhibition; S/CF, shifting/cognitive flexibility. ^∗^*p* < 0.05. ^∗∗^*p* < 0.01.*

## Discussion

Despite increasing understanding of parental influences on child executive functions (Fay-Stammbach et al., 2014), prior research on this topic has rarely controlled for prior levels of child executive functions, included both positive and negative aspects of parenting practices in the same analytic model, and collected data from both mothers and fathers. Moreover, although some 20% of the world population lives in China (United Nations, 2017) and Chinese communities place particular emphasis on child self-regulation (Chen et al., 2012; Kwong et al., 2018), we know next to nothing about whether Chinese parents’ parenting practices may affect the executive functions of their children. This study was the first to examine how mothers’ and fathers’ warmth and hostility were linked to changes in their children’s executive function problems in Chinese families. Partially consistent with our expectations, maternal hostility was linked to increases in child inhibition and shifting/cognitive flexibility problems, and paternal hostility was linked to increases in child updating/working memory problems. On a theoretical level, our findings highlighted the importance of considering the contributions of both mothers and fathers, and differentiating between positive and negative aspects of parenting, in understanding the development of child executive functions. On a practical level, our findings pointed to the utility of targeting maternal and paternal hostility in family intervention and community education in order to reduce child executive function problems.

### The Unique Role of Maternal and Paternal Hostility

The attachment theory posits that optimal parenting allows children to explore their environments and practice their executive functions, without having to constantly monitor their parents’ availabilities and worry that their parents would abandon them (Shaver and Mikulincer, 2010; Bernier et al., 2012). On the other hand, the social learning theory posits that children tend to observe, remember, and reproduce their parents’ behaviors, and that children’s positive and negative development in executive functions may be the result of children’s internalization of their parents’ modeling of good and poor self-regulation, respectively (Hughes and Ensor, 2009; Moriguchi, 2012, 2014). In support of these theories, and previous studies linking maternal (Blair et al., 2011; Kraybill and Bell, 2013; Matte-Gagné et al., 2015; Meuwissen and Englund, 2016) and paternal (Towe-Goodman et al., 2014) warmth in infancy and toddlerhood to child executive function skills in early childhood, our *univariate* analyses demonstrated that parental warmth and hostility were negatively and positively associated with child executive function problems in the following year, respectively. More importantly, expanding on prior work showing that maternal hostility was linked to decreases in child overall skills in executive functions (Cuevas et al., 2014; Devine et al., 2016), our *multivariate* analyses demonstrated that, *controlling for maternal warmth and paternal parenting practices*, maternal hostility was uniquely linked to increases in child inhibition and shifting/cognitive flexibility problems. Moreover, *controlling for paternal warmth and maternal parenting practices*, paternal hostility was uniquely linked to increases in child updating/working memory problems. It is worth mentioning that, by linking parent-reported parenting practices to teacher-reported child outcomes, we addressed some biases due to common method variance (Podsakoff et al., 2003). Furthermore, by controlling for child gender, age, and verbal abilities (as an indicator of general cognitive functioning; Norton and Wolf, 2012; Schipolowski et al., 2014), and maternal and paternal education levels, we ruled out some child and family factors as alternative explanations of our findings. The internal validity of our findings was thus high.

There may be at least two reasons why parental hostility, but not warmth, was uniquely linked to our outcome measures. First, our measure of child executive functions focused on deficiency rather than competence (Gioia et al., 2003; Sherman and Brooks, 2010). Emerging research indicates that the absence of problems does not necessarily indicate the presence of skills, and that child deficiency and skills may be differentially related to family negativity and positivity (Golden and Lashley, 2014; Shulman, 2016). In order to test whether positive and negative aspects of parenting are more closely related to child executive function skills and problems, respectively, future studies should measure both positive and negative aspects of parenting, and both deficiency and competence in child executive functions, to examine the unique associations among them. Second, evidence exists that negative social interactions may be more impactful than positive ones. Research on marital relationships, for example, has established that it takes five acts of spousal love in order to counteract the negative impact of one act of spousal hostility (Gottman, 1993; Holman and Jarvis, 2003). Recent work on parent–child relationships also highlights the importance of maintaining of a high ratio between positive versus negative behaviors of parents toward their children (Zemp et al., 2014). Future research that uses behavioral observations to measure the *relative* frequency of parental warmth versus hostility is needed to examine the differential impact of positive and negative parenting on child executive functions. More generally, however, our findings highlighted the importance of testing the unique impact of positive and negative aspects of parenting in studying the development of child executive functions.

At present, it is hard to produce a definitive explanation as to why maternal and paternal hostility were linked to changes in different executive function problems. However, one possible explanation is that the mother–child relationship may represent a more potent socialization context than the father–child relationship, especially in the early years. Despite the increasing involvement of fathers in childcare in most industrialized societies, mothers continue to spend more time with their children than do fathers (Parke and Buriel, 2006; Lam et al., 2012). Therefore, maternal influences on child development may be more pervasive (Grossmann et al., 2008). In fact, consistent with such views, our bivariate analyses indicated that mothers’ hostility was linked to all three executive function problems in their children, but that fathers’ hostility was only linked to updating/working memory problems in their children. Future researchers should examine whether the extent of parental involvement may moderate the link between parenting and child executive functions.

Another possible explanation is that, regardless of how much time they spend on childcare, mothers and fathers tend to do different things with their children. Data from both the United States (Roeters and Gracia, 2016) and Hong Kong (Kwok et al., 2013) indicate that mothers are more involved in discipline and day-to-day care taking, but that fathers are more involved in play and leisure activities. When parents, often mothers, hostilely ask their children to stop doing something, or to move from one situation or activity to another, their children’s inhibition (i.e., abilities to suppress unthinking or inappropriate thoughts and responses) and shifting/cognitive flexibility (i.e., abilities to shift focus to the most relevant mental framework) may be more affected. On the other hand, when parents, often fathers, express hostility when playing ball games and completing picture puzzles with their children, their children’s updating/working memory (i.e., abilities to hold and manipulate information to achieve goals) may be more affected. As our measure of parental warmth and hostility did not tap onto the social contexts in which these parenting practices had occurred, our data did not allow us to examine these hypotheses. Further studies should test whether the extents of parental involvement in different aspects of child lives, such as discipline versus leisure, may moderate the impact of parenting on child executive functions. On a more general level, however, our findings highlighted the importance of considering the contributions of both mothers and fathers to the development of child executive functions.

Although not the focus of our study, the three subcomponents of executive functions were highly correlated with one another, providing some support to the view that different aspects of executive functions operate in integrated ways to support higher order processing (Garon et al., 2008). In fact, the latent structure of executive functions in early childhood remains an unsolved question in the literature (Lee K. et al., 2013; Chevalier, 2014; Spiegel et al., 2017). However, given that the same teacher provided ratings for multiple children in our study and that the number of clusters (i.e., teachers) was smaller than the number of items in the BRIEF, our data did not allow for a multilevel factor analysis on the outcome measure (Reise et al., 2005; Wright, 2017). Future studies seeking to examine the latent structure of child executive functions should ensure a sufficient case-to-variable ratio, on all levels of analysis, when collecting data.

The practical implications of our findings are nonetheless clear: Through family intervention and community education, mothers and fathers should be informed of the potential negative impact of hostility on their children’s development. Mothers and fathers should also learn how to control angry feelings toward and avert unrestrained conflict with their children. Further, practitioners may consider such methods as mindfulness training (Meppelink et al., 2016) and behavioral coaching (Duncombe et al., 2016) to help mothers and fathers to deal with their hostility when interacting with their children.

### Limitations and Conclusions

This study had several limitations. First, despite our use of longitudinal data, efforts to address common method variance, and consideration of multiple confounding variables, our correlational design limited our ability to draw conclusive remarks on casual relationships. Intervention research that uses randomized experimental designs to manipulate parenting practices and measure subsequent child outcomes is needed to confirm the causal links between parenting and child executive functions. Second, although our sample included families from a wide range of socioeconomic backgrounds, it was not representative of all families with kindergarten children from Hong Kong. Importantly, we did not collect information about whether the participating children had been diagnosed with special needs. Further studies should recruit probabilistic, representative samples, with children with special needs being oversampled, to test the generalizability of our findings to the larger population, as well as to children with special needs. Third, a general lack of studies on parenting and child executive functions with Eastern samples motivated us to conduct ours with Chinese families. More research with non-Western samples is needed. Importantly, although our findings were consistent with some prior work based on European, Canadian, and European American families (Cuevas et al., 2014; Devine et al., 2016), it remains unclear if the *strength* of the relationship between parenting and child executive functions may vary across Eastern and Western communities. It awaits further investigation to use cultural comparative designs to test culture and ethnicity as potential moderators in understanding parental influences on child executive functions (Sabbagh et al., 2006; Ellefson et al., 2017).

Fourth, our analytic models only explained modest to moderate amounts of variance in the outcome measures. This may not be surprising, given prior research showing that child executive functions vary as a function of numerous personal and environmental factors (Cohen, 1988), including brain development, physical fitness, school environments, other family processes (Hughes, 2011; Diamond, 2014; Fay-Stammbach et al., 2014). Additional research should be directed at examining how different personal and environmental factors may jointly affect child executive functions. Finally, our measure of child executive functions was solely based on teachers’ ratings. Since both parents and teachers place great importance on executive functions early on in Chinese communities (Stevenson et al., 1990; Chen et al., 1998; Tobin et al., 2009), their ratings might be correlated due to their own experiences of cultural socialization. In fact, rating measures of executive functions aim more to assess children’s success in goal pursuit in unstructured conditions, and thus are quite different from the more commonly used, performance-based measures, which aim more to assess children’s efficiency in cognitive abilities (Toplak et al., 2012). More generally, considering that teachers’ and parents’ reports, naturalistic and laboratory observations, and structured executive function tasks each provide unique information about children’s executive functions (Garon et al., 2008; Diamond, 2013), future investigators should use multiple methods and multiple sources of information to more comprehensively capture the construct of child executive functions.

In the face of these limitations, our study had important theoretical and practical implications. Theoretically, our findings highlighted the importance of considering both positive and negative aspects of maternal and paternal parenting in understanding the development of child executive functions. Practically, our findings pointed to the utility of helping both mothers and fathers to control their temper at and manage their conflict with their children in order to reduce their children’s executive function problems.

## Ethics Statement

The present study was approved by the Human Research Ethics Committee of the Education University of Hong Kong.

## Author Contributions

All authors contributed to the completion of this manuscript by designing the study, collecting and analyzing the data, and writing up the findings.

## Conflict of Interest Statement

The authors declare that the research was conducted in the absence of any commercial or financial relationships that could be construed as a potential conflict of interest.

## APPENDIX

### Equations for the Multilevel Model of Teachers’ Ratings of Child Executive Function Problems

#### Level 1 (Child-Level) Equation

Child Executive Function Problems*_ij_* = β_0_*_j_* + β_1_*_j_* × Maternal Warmth + β_2_*_j_* × Maternal Hostility + β_3_*_j_* × Paternal Warmth + β_4_*_j_* × Paternal Hostility + β_5_*_j_* × Prior Executive Function Problems + β_6_*_j_* × Child Gender + β_7_*_j_* × Child Age + β_8_*_j_* × Child Verbal Abilities + β_9_*_j_* × Maternal Education + β_10_*_j_* × Paternal Education + *r*_ij_.

#### Level 2 (Teacher-Level) Equations

$$\begin{array}{c} \beta_{0}{}_{j}\;=\;\gamma_{00}+\;u_{0}{}_{j}\\ \beta_{1j}\;=\;\gamma_{10}.\\ \beta_{2j}\;=\;\gamma_{20}.\\ \beta_{3j}\;=\;\gamma_{30}.\\ \beta_{4j}\;=\;\gamma_{40}.\\ \beta_{5j}\;=\;\gamma_{50}.\\ \beta_{6j}\;=\;\gamma_{60}.\\ \beta_{7j}\;=\;\gamma_{70}.\\ \beta_{9j}\;=\;\gamma_{90}.\\ \beta_{10j}\;=\;\gamma_{100}. \end{array}$$
